# News and Coming Events

**Published:** 1908-01-04

**Authors:** 


### NEWS AND COMING EVENTS.

A special ward for electric treatment has recently been opened in the Northern Infirmary.

The next session of the West London Postgraduate College will commence on Monday, January 13th, 1908. The session lasts twelve weeks.

It has been decided to rebuild completely the Ulster Hospital for Women and Children at Mountpottinger. For this purpose a sum of £10,000 is required.

Dr. George F. Shrady, founder of the New York *Medical Record*, died at his residence in New York on November 29 last. Dr. Shrady was for thirty-eight years chief editor of the *Record*, and was one of the foremost of American medical journalists.

According to the *Tidsschrift for Kemi og Farmaci*, the oldest medical document in the ancient Norse language is the *Icelandic Pharmacopœia*, published in the thirteenth century. It now forms part of a collection of antique manuscripts in the Royal Library of Copenhagen. The work consists of five octavo pages and was intended as a guide to the people in times of sickness and accident. Needless to say, the methods of treatment were primitive in the extreme.

At the annual meeting of the Christie Hospital (Cancer Pavilion and Home) in Upper Brook Street, Manchester, held on December 5 last, Professor R. B. Wild stated that the medical officers of this institution had tried all the advertised remedies which had been at all prominent in the papers for the last ten years. They would not, however, try any secret remedy, because they did not know what harm might arise. Up to the present time no adequate solution whatever had been found for the cause of cancer. He did not think that the last one, which was being much boomed at present—that tea and coffee were the most probable cause of cancer—was at all likely to be any better than its predecessors. Books of that kind, which were boomed for a time, were apt to mislead people and do a considerable amount of harm.

At the same meeting, Sir William Sinclair, one of the honorary physicians, stated that he did not believe for a moment that pure genuine cancer could be produced by inoculation. One of the most distinguished of American surgeons had assured him that he had inoculated his own arm with the most corrupt piece of cancer he could find, and he was none the worse for it one or two years afterwards.

Hospital Saturday Fund.—The annual dinner of the Association will be held at the Holborn Restaurant on Saturday, February 1, 1908, at 6.30 p.m. The chair will be taken by Sir George Bartley, K.C.B., one of the vice-presidents of the Fund, who will be supported by the honorary officers of the Fund and other gentlemen. The Dinner Committee cordially invite all members of the Board of Delegates and of local committees and all ladies and gentlemen interested in the work of the Fund to attend this social re-union. The wearing of evening dress will be optional. An attractive programme of music, etc., will be provided by the committee. Applications for tickets, which are 4s. 6d. each, should be made to the Secretary of the Fund, or the chairman or hon. secretary of any of the standing committees, not later than Tuesday, January 28, 1908.

Dr. Henry Willson, of Weybridge, has been placed upon the Commission of the Peace for the County of Surrey.

Professor T. Annandale, of Edinburgh, died suddenly on Friday morning, December 20. The death is also announced of Sir Patrick Heron Watson, at Edinburgh, on December 21.

The preliminary programme of the next International Medical Congress has been issued. The Congress will be held in Budapest, August 29 to September 4, 1909, under the patronage of Emperor Francis Joseph I. Twenty-one sections are provided for, as well as two general sessions at the opening and closing of the Congress. The manuscript of papers to be read must be in the hands of the secretary of the Congress by January 31, 1909. Correspondence is to be directed to the office of the Sixteenth International Medical Congress, Budapest, VIII., Esterhazy-Utcza, 7.

The only member of the profession whose name appears in the New Year's honour list, apart from decorations for surgeons of the Indian Services, is Mr. Malcolm Morris, on whom the Knight Commandership of the Victorian Order is conferred. Best known as the author of the most widely-read of the smaller treatises on diseases of the skin, the new knight is Dermatologist to King Edward VII. Hospital and to the Seamen's Hospital, and Consulting Surgeon to the skin department of St. Mary's Hospital, as well as author of many monographs and articles dealing with his specialty. The congratulations of the profession are due to him on the occasion of this well-deserved honour.

